# hnRNPA2 mediated acetylation reduces telomere length in response to mitochondrial dysfunction

**DOI:** 10.1371/journal.pone.0206897

**Published:** 2018-11-14

**Authors:** Manti Guha, Satish Srinivasan, F. Bradley Johnson, Gordon Ruthel, Kip Guja, Miguel Garcia-Diaz, Brett A. Kaufman, M. Rebecca Glineburg, JiKang Fang, Hiroshi Nakagawa, Jeelan Basha, Tapas Kundu, Narayan G. Avadhani

**Affiliations:** 1 Department of Biomedical Sciences, School of Veterinary Medicine, University of Pennsylvania, Philadelphia, PA, United States of America; 2 Department of Pathology and Laboratory Medicine, Perelman School of Medicine, University of Pennsylvania, Philadelphia, PA, United States of America; 3 Penn Vet Imaging Core, School of Veterinary Medicine, University of Pennsylvania, Philadelphia, PA, United States of America; 4 Department of Pharmacological Sciences, Stony Brook University, Stony Brook, NY, United States of America; 5 Vascular Medicine Institute, University of Pittsburg, Pittsburgh, PA United States of America; 6 Department of Gastroenterology, Perelman School of Medicine, University of Pennsylvania, Philadelphia, PA, United States of America; 7 Transcription and Disease Laboratory, Molecular Biology and Genetics Unit, Jawaharlal Nehru Centre for Advanced Scientific Research, Jakkur, Bangalore, India; Centre National de la Recherche Scientifique, FRANCE

## Abstract

Telomeres protect against chromosomal damage. Accelerated telomere loss has been associated with premature aging syndromes such as Werner’s syndrome and Dyskeratosis Congenita, while, progressive telomere loss activates a DNA damage response leading to chromosomal instability, typically observed in cancer cells and senescent cells. Therefore, identifying mechanisms of telomere length maintenance is critical for understanding human pathologies. In this paper we demonstrate that mitochondrial dysfunction plays a causal role in telomere shortening. Furthermore, hnRNPA2, a mitochondrial stress responsive lysine acetyltransferase (KAT) acetylates telomere histone H4at lysine 8 of (H4K8) and this acetylation is associated with telomere attrition. Cells containing dysfunctional mitochondria have higher telomere H4K8 acetylation and shorter telomeres independent of cell proliferation rates. Ectopic expression of KAT mutant hnRNPA2 rescued telomere length possibly due to impaired H4K8 acetylation coupled with inability to activate telomerase expression. The phenotypic outcome of telomere shortening in immortalized cells included chromosomal instability (end-fusions) and telomerase activation, typical of an oncogenic transformation; while in non-telomerase expressing fibroblasts, mitochondrial dysfunction induced-telomere attrition resulted in senescence. Our findings provide a mechanistic association between dysfunctional mitochondria and telomere loss and therefore describe a novel epigenetic signal for telomere length maintenance.

## Introduction

Telomeres protect the chromosome ends and are important in cellular defense against inducers of DNA damage such as xenobiotic chemicals and radiation [1–3]. Telomere length is maintained at equilibrium and progressively shortens during replication [4]. Loss of telomeres results in chromosomal abnormalities, typical of tumor cells and senescence. Cellular aging is associated with shortened telomeres, and associated risk for chromosomal damage, which is also associated with pathologies including cancer [1,5,6]. Multiple mechanisms have been proposed for the maintenance and regulation of telomere length under pathological or cellular stress conditions. Telomere length is maintained by a balance between telomere shortening processes such as DNA replication and telomere lengthening factors such as ribonucleoprotein telomerase, telomere binding proteins and telomere capping proteins [7]. While some studies suggest that epigenetic regulation of telomeric histones contributes to telomere homeostasis [8,9], the complex mechanisms involved in telomere maintenance under pathophysiological conditions remain unclear.

Mitochondria are highly susceptible to damage from numerous factors including free radicals, environmental chemicals, radiation exposure, and lipid peroxides produced by defective electron transport chain, the hypoxic environment prevalent in solid tumors and defective mtDNA transcription and replication machinery [10–13]. These cellular and environmental stressors can cause mitochondrial defects such as reduction in mtDNA copy number, mtDNA mutations/deletions and impaired electron transport chain activity. In fact, defects in OXPHOS and accumulating mtDNA mutations are associated with aging and age-associated cancers. A common pathological feature of both of these diseases is shortened telomere DNA leading to DNA damage [6]. A recent study demonstrated that mtDNA haplotype influences mitochondrial dysfunction and associated aging parameters such as telomere attrition in conplastic mice [14].

Some reports suggest the contribution of dysfunctional mitochondria to telomere maintenance in human pathologies. In a breast cancer cell line, suppression of telomerase component, hTERT resulted in telomere shortening and mitochondrial dysfunction including reduced mtDNA content [15]. Interestingly, rescuing mitochondrial functions by overexpressing MnSOD, was sufficient to ameliorate the telomere length shortening and associated phenotype [15]. Mitochondrial dysfunction induced by SOD2 deficiency is associated with aging phenotype in skin [16]. In Duchene Muscular Dystrophy associated cardiomyopathy, telomere loss is associated with mitochondrial fragmentation and treatment with antioxidants, rescues mitochondrial functions as well as the telomere length suggesting a link between oxidative stress and telomere length [17]. While these observations have led to a unified theory of aging and cancer associated with telomere shortening, it remains unclear how dysfunctional mitochondria in tumor and aging cells play a causal role in telomere dynamics.

Our previous studies showed that mitochondrial dysfunction activates a mitochondria-to-nucleus stress response, which is a cellular adaptation to mitochondrial stress [18–22]. We demonstrated that altered cytosolic calcium and activation of calcineurin are upstream signals for the propagation of the mitochondrial stress response, which reprograms immortalized cells to a tumorigenic phenotype [18–24]. We also showed transcriptional upregulation and activation of heterogeneous ribonuclear protein (hnRNP) A2 in response to mitochondrial dysfunction. We demonstrated further that hnRNPA2 functions as a transcriptional coactivator of mitochondrial stress activated nuclear genes [25–27]. More recently, we reported that hnRNPA2 is a histone lysine acetyltransferase (KAT), which mediates H4K8 acetylation on target gene promoters [28]. HnRNPA2-mediated acetylation is critical for nuclear gene activation including that of telomerase, and overall propagation of mitochondrial stress response and acquired invasiveness in C2C12 cells.

In this study our goal was to understand the underlying mechanism by which mitochondrial dysfunction modulates telomere length. We demonstrate here that dysfunctional mitochondria activate hnRNPA2 which in turn acetylates telomeric histones at H4K8. Using loss and gain-of-function studies, we demonstrate that H4K8 acetylation is a signal for telomere shortening in response to mitochondrial stress. Notably, hnRNPA2 plays a dual role in telomeric H4K8 acetylation and shortening of telomere length and telomerase activation through H4K8 acetylation of promoter region. In stressed cells, therefore, telomere shortening occurs despite increased telomerase expression.

## Materials and methods

### Cell lines and plasmids

Murine skeletal myoblast C2C12 cells were purchased from ATCC (CRL 1772). We used isogenic murine C2C12 cells for telomere length comparisons. We compared parental C2C12 cells, C2C12 cells in which mitochondrial DNA was depleted by using either ethidium bromide (100 ng/ml) or 2,3′-dideoxycytidine (10 μM, 120 h) or expressing *Tfam* shRNA (mtDNA Depl). We also used C2C12 cells in which mitochondrial DNA content was restored to 80% of parental cells by culturing in the absence of inhibitors (reverted cells). We earlier reported that mtDNA-depleted C2C12 cells exhibit higher proliferation compared to parental cells [20,25,29].To exclude the possible proliferative differences on telomere length, we used cells from identical passage numbers for assaying mitochondrial function and telomere length.

C2C12 cells were genetically modified using hnRNPA2 shRNA and/or expression of WT or KAT mutant hnRNPA2 constructs as described below: Three independent shRNA constructs targeted to hnRNPA2 mRNA were validated in preliminary experiments as reported earlier [25] and one shRNA target was selected for generating stably expressing mtDNA-depleted/hnRNPA2shRNA cell line [25]. As described before [28] mtDNA-depleted C2C12 cells stably expressing shRNA (cloned in pLKO.1 vector) against either hnRNPA2 or GFP (negative control) were used in this study. Full-length cDNA for human hnRNPA2 cloned in pET28a (+) vector with N-terminal 6x His tag was a gift from Gideon Dreyfuss (University of Pennsylvania). Full length 6xHis-hnRNPA2/pET28a(+) with Arg48Thr, Arg50Thr substitutions were generated using the Quick Change Lightning site-directed mutagenesis kit (Agilent technologies). Expression and purification of the recombinant 6xHis-hnRNPA2 proteins were reported earlier [28]. For *in vivo* studies in C2C12 and other cells, the WT and mutant hnRNPA2 constructs were subcloned in pMXs vector (a kind gift from Dr. Russ Carstens, UPENN). For reconstitution of hnRNPA2 knock down cells with WT and mutant hnRNPA2 proteins, the cDNA constructs carrying conservative mutations at the shRNA target region were introduced in the hnRNPA2 knock down cells. For generating stably expressing WT and mutant cDNAs, mtDNA-depleted/hnRNPA2sh expressing cells were transduced with either pMXS-IRES- Puro- EGFP empty vector or KAT mutant cDNAs. The reconstitution with appropriate cDNA was confirmed by western immunoblot as described earlier [28].

For *Tfam* silencing experiments, *Tfam* and GFP shRNAs cloned in pLKO.1 lentiviral vectors were used. Five independent *Tfam* shRNA constructs were used for initial screening experiments and cells stably expressing *Tfam* shRNA were generated using puromycin (2.0μg/ml) selection. Cells expressing *Tfam* shRNA were maintained in medium supplemented with uridine (50 μg/ml) and sodium pyruvate (1mM).

### Animals

The MPV17 knock out mice used in this study were obtained from Jackson Laboratories (CFW-Mpv17/J, JAX stock #002208) [30] and bred to BALB/c mice for 10 generations. All animals used in this study were age matched (6 weeks). Animals were housed and cared for in accordance with the regulations of the University of Pennsylvania’s Institutional Animal Care and Use Committee. Mice were euthanized using CO_2_ asphyxiation using an IACUC approved protocol before harvesting tissues.

### Mean telomere length analysis

Mean telomere length was measured by real-time PCR [31] and also by in-gel hybridization with a radiolabeled telomere repeat probe [32] as well as by Fluorescent *In Situ* hybridization (telo-FISH). Telomere length analysis by real-time PCR was conducted as previously described from total DNA in control, mtDNA-depleted, and mtDNA-depleted/hnRNPA2sh C2C12 cells using specific primers for mouse telomeric repeats.

The mouse-specific single copy gene acidic ribosomal phosphoprotein P0 (36B4) was used as an internal control for normalization. In this quantitative PCR based method, we compared relative differences in the mean telomere length between isogenic C2C12 cell lines to test if telomere length was altered as a consequence of experimentally induced mitochondrial dysfunction.

Telomere length was also analyzed in parallel by PFGE (1.2% agarose gel at 6 V/cm at 15ºC for 20 hours with switch times from 1-12s) and in-gel hybridization [33]. Dried gels were treated with NaOH to denature the DNA, hybridized with a ^32^P end-labeled (CCCTAA)_4_ probe, and then visualized by autoradiography.

Quantitative Fluorescent *In Situ* hybridization (Q-FISH) was performed with Cy3–telomere PNA probe as described before [33,34]. Relative Cy3 intensity normalized to the DAPI intensity was used as a measure of total telomere length per nucleus.

#### Metaphase chromosome preparation, Q-FISH and image analysis

Cells were treated with 0.1μg/ml Colcemid solution (Sigma, St. Louis, MO) for 3 hours and harvested for metaphase spreads. Cells were swollen in hypotonic solution at 37°C for 15 minutes, and fixed with methanol: acetic acid (3:1) with three repeated exchanges prior to dropping onto slides and dried overnight.

Nuclei and metaphase spreads were processed for telomere Q-FISH. After washing and hypotonic swelling, cells were fixed and stored in methanol/acetic acid fixative using standard procedures [35]. Nuclei and metaphase spreads were fixed on slides. The slides were dried overnight in air and immersed in Phosphate Buffered Saline (PBS) for 5 min prior to fixation in 4% formaldehyde in PBS for 2 min; slides were then washed in PBS (3 × 5 min) and treated with pepsin (Sigma, St. Louis, MO) at 1 mg/ml for 10 min at 37°C, pH 2.0. After a brief rinse in PBS, the formaldehyde fixation and washes were repeated and the slides were dehydrated with ethanol and air-dried. Hybridization mixture containing 70% formamide, Cy3 PNA probe (Cy3-OO-CCCTAACCCTAACCCTAA), and 1% (W/V) blocking reagent in 10 mM Tris pH 7.2 was added to the slide, a coverslip (20 × 20mm) was added and DNA was heat denatured. After hybridization for 2h at room temperature, the slides were washed at room temperature with 70% formamide/10 mM Tris pH 7.2 (2 × 15 min) and with 0.05 M Tris 0.15 M NaCl pH 7.5 containing 0.05% Tween-20 (3 × 5 min). The slides were then dehydrated with ethanol, air-dried and covered with Aquamount solution (Thermo Scientific, Philadelphia PA) containing 0.1 μg/ml of DAPI.

The nuclei and metaphases on the PNA hybridized slides were visualized under a Nikon microscope and images were captured under 100x objective. The image acquisition conditions were kept identical for all cell types. For quantitation, the raw images of nuclei were used for analysis using MetaMorph software (Molecular Devices). Cy3-PNA signals were counted and the fluorescence intensity was quantitated by applying consistent intensity and size thresholds. The average DAPI fluorescence intensity for each nucleus was quantified and used to normalize the measured Cy3 PNA fluorescence intensities. The total DAPI fluorescence signal for each nucleus was quantified. At least 10 nuclei were counted for each cell type.

#### Immunofluorescence staining and telomere FISH of metaphase spreads (meta-TIF assay)

Meta-TIF assays were performed as described in Cesare et al [36,37]. Briefly, cells were treated with colcemid (0.1μg/ml, 2h), trypsinized and resuspended in hypotonic buffer containing 0.2% (w/v) KCl and 0.2% (w/v) trisodium citrate for 10 min at room temperature. For obtaining metaphases, cells were cytocentrifuged at 450*g* for 10 min onto a positively charged super frost glass slides. Metaphase preparations on the glass slides were fixed in 4% formaldehyde in PBS for 10 min at room temperature, permeabilized for 10 min at room temperature in KCM buffer (120 mM KCl, 20 mM NaCl, 10 mM Tris (pH 7.5) and 0.1% (v/v) Triton X-100) and blocked with 100 μg ml^−1^ DNase-free RNase A (Sigma) in antibody dilution buffer (20 mM Tris (pH 7.5), 2% (w/v) BSA, 150 mM NaCl, 0.1% (v/v) Triton X-100 and 0.1% (w/v) sodium azide) for 15 min at 37°C. Primary H4K8ac antibody was diluted 1:100 in antibody dilution buffer for 1 h at room temperature, then washed in 1× PBST (1× PBS with 0.1% (v/v) Tween-20), and incubated for 30 min at room temperature in secondary antibody conjugated to Alexa 488 (1:1000 dilution). For proceeding with the FISH, the metaphases slides were fixed in 4% formaldehyde in PBS for 10 min at room temperature. The slides were dehydrated in 70%, 90% and 100% ethanol and air dried. The dehydrated slides were incubated with Cy3 PNA probe (Cy3-OO-CCCTAACCCTAACCCTAA) in PNA hybridization solution (70% (v/v) deionized formamide, 0.25% (v/v) Roche blocking reagent, 10 mM Tris (pH 7.5), 4 mM Na_2_HPO_4_, 0.5 mM citric acid and 1.25 mM MgCl_2_), incubated at 80°C for 3 min and hybridized them at room temperature for 2 h. The slides were washed in PNA wash A (70% (v/v) formamide and 10 mM Tris (pH 7.5)), washed them in PNA wash B (50 mM Tris (pH 7.5), 150 mM NaCl and 0.08% (v/v) Tween-20). The slides were rinsed in deionized water and mounted using Gold Antifade containing DAPI. Images were captured on Leica widefield microscope under 100x objective.

### Chromatin Immunoprecipitation (ChIP) analysis

ChIP assays were performed as previously described [25,28]. Primer sequences used for quantitative real time PCR amplifications are provided below.

### Antibodies

Acetyl-Histone H4 (Lys8): Millipore, Billerica, MA; Cat # 17–10099

Anti-Histone H4 antibody: Millipore, Billerica, MA; Cat # 05-858R; clone 62-10C-2

Anti-hnRNPA2 antibody: Santa Cruz Biotechnology, Dallas, TX; Cat # sc-32316, clone # DP3B3

Anti- beta actin antibody: Santa Cruz Biotechnology, Dallas, TX; Cat # sc-47778, clone # C4

Anti-53BP1 antibody: Abcam, Cambridge, MA; Cat # ab36823

Anti-gamma H2A.X antibody: Abcam, Cambridge, MA; Cat # ab2893

### Primer sequences

#### Telomere PCR

**Telomere DNA primers**:

G-rich: GTTTGTTTGGGTTTGGGTTTGGGTTTGGGTTTGGGTT

C-rich: GGCTTGCCTTACCCTTACCCTTACCCTTACCCTTACCCT

**Internal control primers**:

36B4 Forward Primer: ACT GGT CTA GGA CCC GAG AAG

36B4 Reverse Primer: TCA ATG GTG CCT CTG GAG ATT

#### Telomere-FISH

**Cy3 PNA probe:** Cy3-OO-CCCTAACCCTAACCCTAA

### Statistical analysis

All data are representative of at least 3 independent experiments. Statistical significance was determined using analysis of variance (ANOVA). A p < 0.05 was considered statistically significant. * indicates p< 0.05.

## Ethics statement

This study was carried out in strict accordance with the guidelines for animal research in the University of Pennsylvania’s Institutional Animal Care and Use Committee (IACUC) and the NIH. The protocol (Protocol Number: 805731) was approved by the Institutional Animal Care and Use Committee (IACUC). Mice were euthanized using CO_2_ asphyxiation following IACUC approved protocol and Guideline for the Care and Use of Laboratory Animals of the National Institutes of Health. Tissues were harvested only after euthanasia.

## Results

### Impairment of mitochondrial functions induces telomere shortening

**Fig 1:**
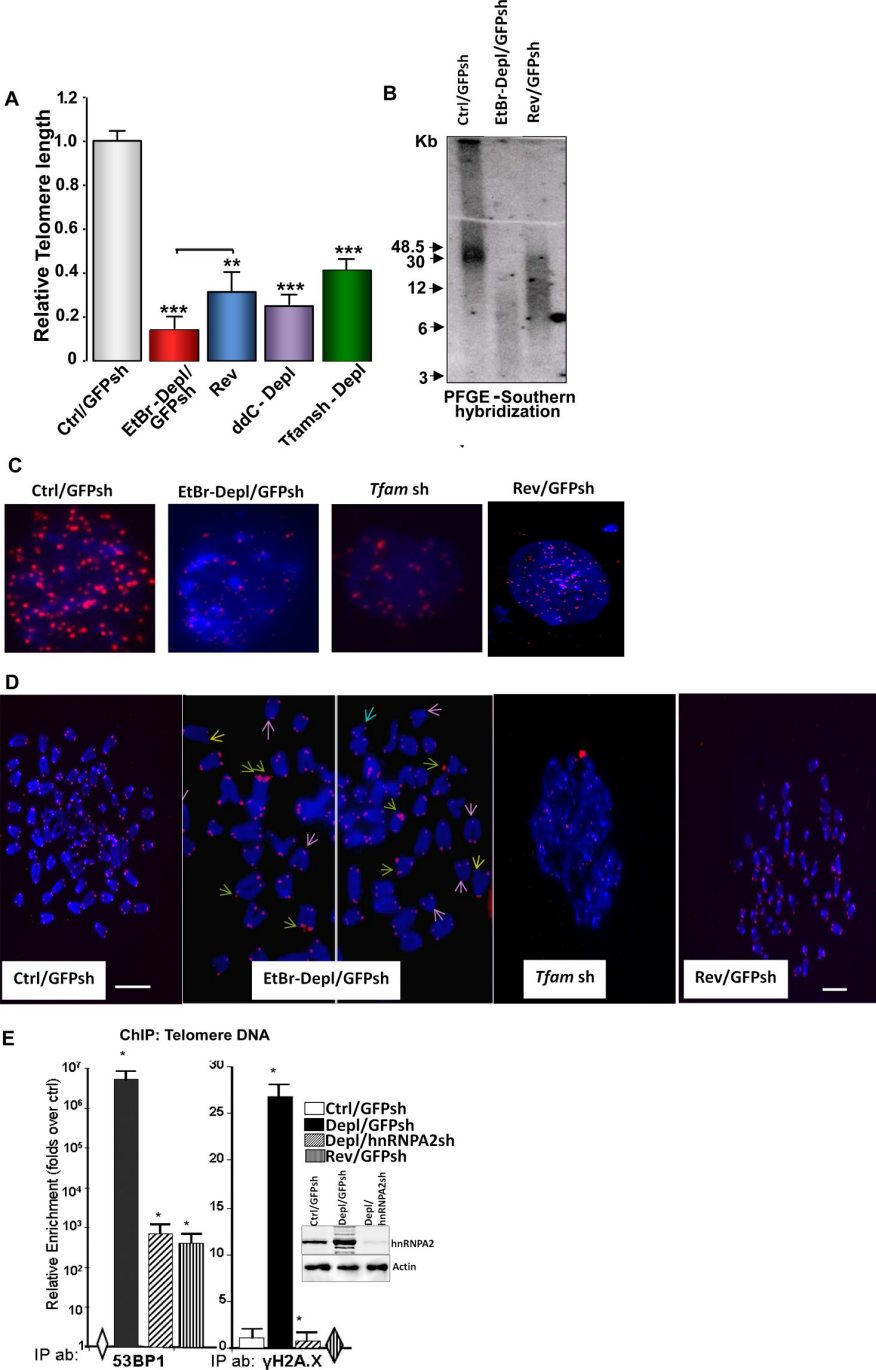
Partial mtDNA depletion-induced telomere length attrition in immortalized C2C12 cells. (A) Relative Telomere length analysis using genomic DNA isolated from control, mtDNA-depleted (EtBr treated, 2,3’-ddC treated or *Tfam* shRNA as indicated in the figure) and reverted cells. Real Time PCR was performed using primers specific for telomere DNA and normalized using control DNA (36B4.Primer sequences are provided under Materials and Methods; **(B)** PFGE of total DNA from control, mtDNA-depleted, and reverted cells followed by in-gel hybridization using a ^32^P-labeled telomere DNA-specific probe. **(C)** Q-FISH on nuclei of control, MtDNA-depleted (EtBr or Tfam sh) and reverted cells. Telomeres are probed with Cy3-PNA (red) and nucleus stained with DAPI (blue). The width of each field shown is 15 μm. **(D)** Representative image of Q-FISH of telomere Cy3-PNA probe (Red) on metaphase spreads (Blue) in Control, mtDNA depleted (EtBr or *Tfam* sh) and reverted cells. Fragile telomeres (green arrows), signal-free ends (yellow arrows) and chromosome end fusions (pink arrows) are indicated in cells depleted of mtDNA. Chromosomes were stained with DAPI (blue). Scale bar = 15 μm for all panels. **(E)** DNA Damage Response Activated in MtDNA depleted cells. Recruitment of DNA damage response factors at the telomere DNA assessed by ChIP analysis in control, MtDNA-depleted, MtDNA-depl/hnRNPA2sh and reverted C2C12 cells. Immunoprecipitations were performed using antibodies to DNA damage response factors, 53BP1, and phosphorylated H2A.X. Western Immunoblot in control, mtDNA depleted and mtDNA-depleted/hnRNPA2sh cells shows the efficiency of hnRNPA2 shRNA.

Recently we showed that partial mtDNA depletion (about 70–80%) in C2C12 and other cells induces telomerase activation and a highly proliferative phenotype [28]. In this study we investigated telomere length in control, mtDNA-depleted (EtBr-Dep treated) and reverted C2C12 cells by complimentary approaches including real-time PCR [31], Southern hybridization of telomeric DNA separated from internal sequences using ^32^P labeled C-rich telomeric DNA probe and telomere Q-FISH analysis. Fig 1A shows that relative telomere length was markedly reduced in C2C12 cells treated with ethidium bromide (EtBr/ GFPsh) or ddC for mtDNA depletion. In both cases, the relative telomere length was reduced by 55–70%. In cells in which mtDNA was partially restored (i.e. reverted cells), the telomere length was partially rescued compared with the EtBr depleted cells (Fig 1A). We utilized a genetic mode of mtDNA depletion by stable expression of *Tfam* shRNA which resulted in reduction of mtDNA copy numbers (Figure A in S1 File). Similar to mtDNA depletion by EtBr, *Tfam* shRNA mediated mtDNA depletion also resulted in the activation of calcineurin Aα (CnAα) and hnRNPA2, important markers of the mitochondrial retrograde stress (MtRS) signal (Figure B in S1 File) and also resulted in telomere length reduction although it was less severe (Fig 1A). These results together suggest that telomere shortening was related to mitochondrial stress response.

Resolution of telomere DNA in control, mtDNA depleted (EtBr treated) and reverted C2C12 cells by pulse field gel electrophoresis followed by hybridization with C-rich ^32^P-end labeled DNA probe also yielded a similar pattern in that there was marked reduction in telomere size/mass which was restored in reverted cells (Fig 1B). To rule out the effects on EtBr treatment in our observed reduction in telomere length in mtDNA-depleted cells, we analyzed telomere size /mass by in-gel hybridization in C2C12 cells where mtDNA was depleted using alternative chemical and genetic approaches (2’,3’-ddC treatment, *Tfam* shRNA) and observed marked reduction in telomere content in mtDNA-depleted cells compared to control or reverted cells (Figure C in S1 File). Telomere Q-FISH analysis showed marked loss of telomere signal intensities in mtDNA-depleted cells (Fig 1C). Another striking outcome of mtDNA depletion was a high incidence of telomeric aberrations with signal-free ends at the chromatids and marked number of chromosome end-fusions (Fig 1D). A large number of chromatids in mtDNA-depleted cells showed multiple telomere signals at each chromatid end (Fig 1D) characteristic of “fragile” telomeres [34]. These results together show a marked reduction in relative telomere size coupled with fragile ends in cells subjected to mitochondrial stress. Use of three parallel approaches also show that results with Q-PCR method used with appropriate controls mirrors the data obtained with other two methods. We therefore used the Q-PCR method in subsequent experiments.

Shortening of telomeres leads to telomere uncapping, chromosomal instability and activation of a DNA damage response (DDR) [1,3,12,38]. Uncapped telomere ends recruit DDR factors 53BP1 and phosphorylated H2A.X (γH2AX). Both 53BP1 and γH2A.X were enriched at telomeres in mtDNA-depleted C2C12 cells indicative of uncapping and chromosomal DNA damage. We observed reduced association of these DDR factors in reverted and hnRNPA2 silenced cells (Fig 1E). In our chromatin immunoprecipitation (ChIP) assay, we observed differences between 53BP1 and γH2A.X in the relative enrichment of these factors at the telomeres of the mtDNA-depleted, reverted and mtDNA-depl /hnRNPA2 silenced cells when compared to the control cells. This difference between the two DDR factors in fold enrichment could be attributed to: *1*. Difference in the affinities of the two antibodies and/or, *2*. The level of 53BP1 is undetectable while there is low level of γH2A.X in control cells and our method of estimation of relative fold enrichment is compared over control cells which could possibly reflect on the relative values. Important to point out that the pattern of enrichment of both factors remains similar in mtDNA depleted cells. Moreover, while γH2AX level in the mtDNA-reverted and depl/hnRNPA2 silenced cells is similar to that of control cells, we observed that the 53BP1 levels in reverted and depl/hnRNPA2 silenced cells are markedly lower than in mtDNA depleted cells which do not fully revert to that of the control cell level. Even though 53BP1 and γH2A.X are both markers of DNA damage, they respond to different signal inputs and therefore not necessarily respond identically to the stress signals from our genetic manipulations. Moreover, we suspect even under partial reversal of mitochondrial stress in our reverted cells, there remains possibly low levels of DNA damage accounting for our detection of 53BP1 in reverted and hnRNPA2 silenced cells. Our results suggest a link between telomere attrition and activation of the DNA damage response at telomeres in response to mitochondrial dysfunction. It is well established that DNA damage induces telomere shortening independent of mitochondrial stress [1,2]. To ascertain the distinctive nature of the mitochondrial stress-induced telomere attrition, we induced nuclear DNA damage by zeocin treatment (Figure A in S2 File). As expected, zeocin treatment caused telomere shortening but did not induce the expression of mitochondrial stress marker gene hnRNPA2 (Figure B in S2 File). This suggests that telomere shortening in mtDNA depleted cells and zeocin mediated DNA damage involve distinctly different mechanisms.

### Shortened telomeres in MPV17 KO mouse model of mtDNA depletion

**Fig 2:**
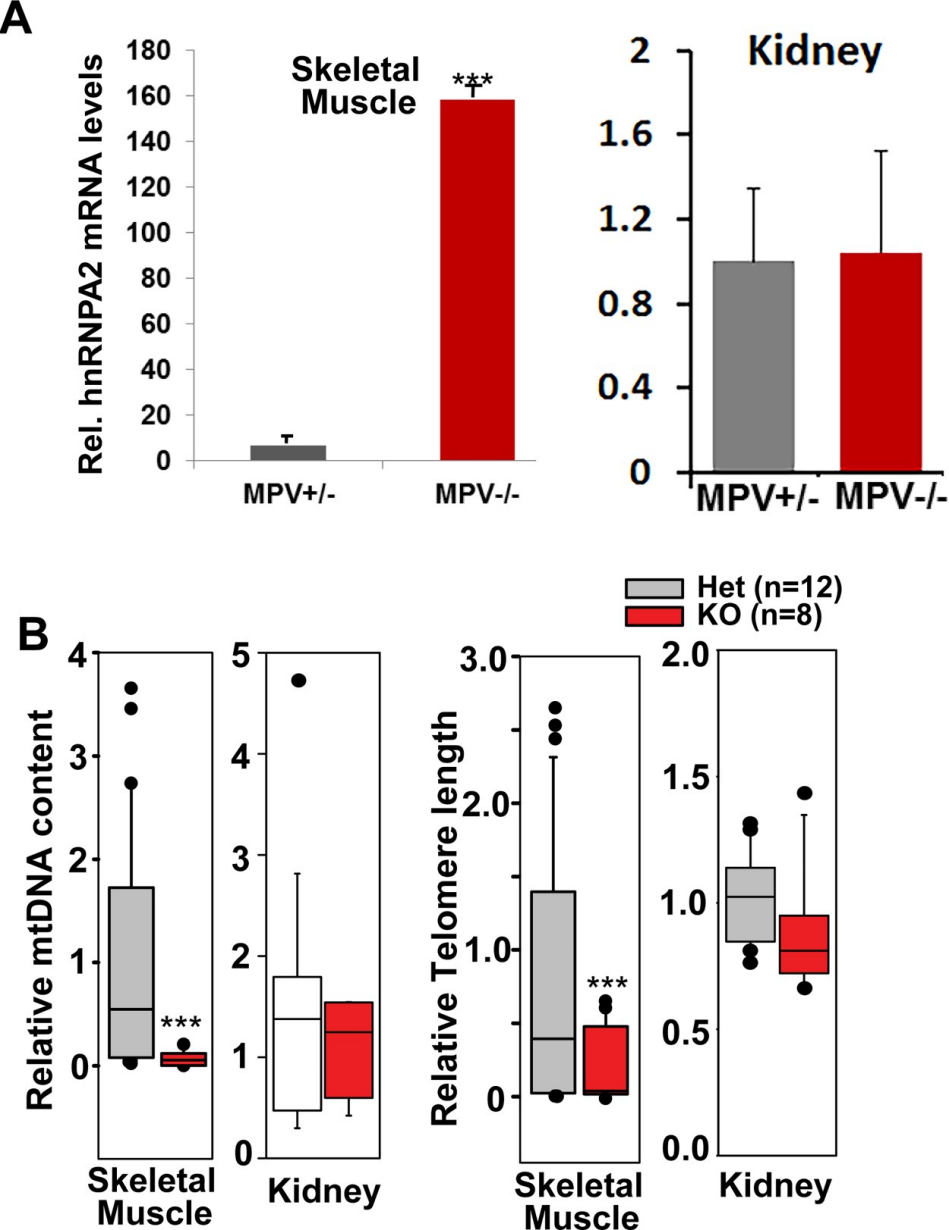
MtDNA depletion induced telomere shortening in vivo. **(A)** Real time PCR showing mRNA levels of hnRNPA2 in skeletal muscle and kidney tissues in MPV +/- and MPV-/- mice; n = 12 for MPV +/- and n = 8 for MPV-/- mice. **(B)** Box plots of telomere length and mtDNA content for skeletal muscle and kidney tissues of MPV17 +/-and MPV-/- mice; n = 12 for MPV+/-, n = 8 for MPV-/- mice. The line within each box represents the median. Data are represented as mean ± SD.

MPV17 is a mitochondrial inner membrane protein whose loss of function impairs oxidative phosphorylation and results in severe mtDNA depletion syndrome in humans and in MPV17^−/−^ mice [39]. In animal models, homozygous loss of MPV17 exerts tissue-specific effects and more deleterious effects on mtDNA content are observed in liver and skeletal muscle with negligible effects on brain and kidney [39,40]. Notably MPV17^+/-^ heterozygous mice exhibit no reported mitochondria-related phenotype and are similar to MPV17^+/+^ mice [40]. We therefore used skeletal muscle and kidney tissues from MPV17 KO mice to study the effects of varying degrees of mtDNA depletion on telomere length maintenance (Fig 2). Similar to our observations in C2C12 skeletal myocytes, we observed an induction of hnRNPA2 in skeletal muscle (Fig 2A, left panel) of MPV17^-/-^ mice, but not in the kidney (Fig 2A, right panel) of homozygous knock out mice. HnRNPA2 is a key marker of mitochondrial stress response [25,28]. Results show that compared to the MPV17^+/-^, the mtDNA content of the MPV17^-/-^ skeletal muscle is markedly lower (Fig 2B). In kidneys of the MPV17^-/-^ mice, the mtDNA depletion is considerably lower than in the muscle (Fig 2B). In accordance, the skeletal muscle of MPV17^-/-^ mice exhibited shorter telomeres while the kidneys showed no significant reduction in telomere length (Fig 2B). These results essentially validate the results of telomere loss shown in C2C12 cells. These results also support the possibility that mtDNA loss is a causal signal for telomere shortening.

### Generality of telomere attrition in different cells and by different stress modalities

**Fig 3:**
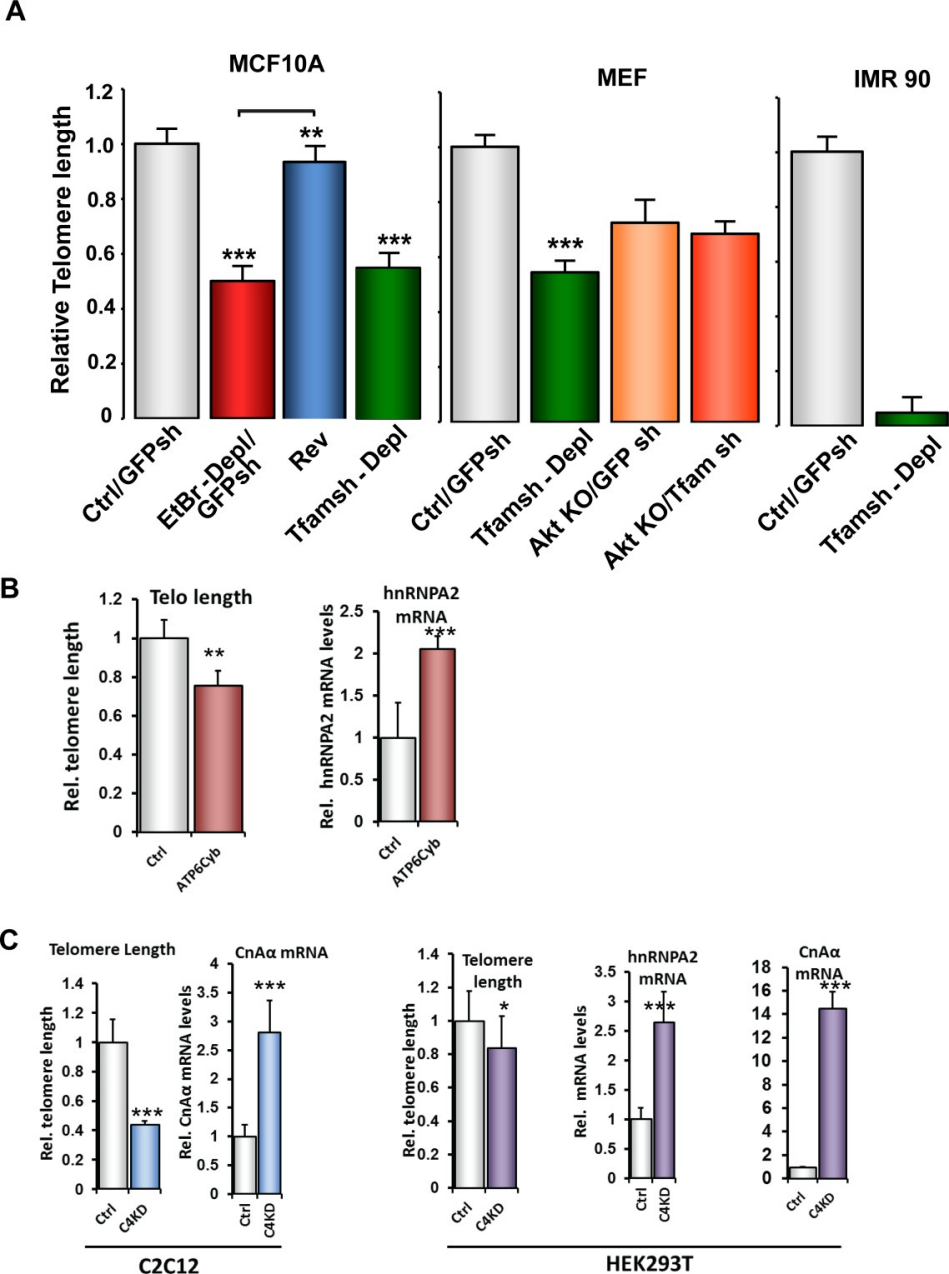
Telomere length attrition induced by alternate approaches of mitochondrial dysfunction. **(A)** Telomere length in control and MtDNA-depleted MCF10A, MEF and IMR90 cells assessed by real time PCR. **(B)** Relative telomere length and hnRNPA2 mRNA levels in 143BTK- control and ATP6 cybrid cells assessed by real time PCR. **(C)** Relative telomere length and transcript levels of MtRS genes (CnAα and hnRNPA2) in control and CcOIVi1KD C2C12 and HEK293T cells.

To test the generality of the telomere shortening in response to mtDNA depletion we tested mtDNA depleted MCF10A and MEF and IMR90 cells (Fig 3). We observed that mtDNA-depletion in MCF10A, MEF and IMR-90 cells either by EtBr treatment or by silencing *Tfam* mRNA, which we previously reported activates MtRS [18,20,22,25,28,41] resulted in similar reduction in telomere length and by restoring the mtDNA content, telomere length was restored (Fig 3A). These results indicate a causal role of mtDNA depletion on telomere attrition and suggest that 1) the effect is not cell-type specific and 2) the effect is not due to toxic side effects of EtBr binding to telomere DNA since mtDNA depletion by *Tfam* mRNA depletion also yielded similar effects. We previously reported that Akt1 is activated in response to mtDNA depletion [27]. We observed telomeres were shorter in Akt knockout MEFs (Fig 3A, middle panel: MEF cells). Notably mtDNA depletion in these cells had no further effect on telomere shortening which is in agreement with our previous report that Akt1 silencing abrogates MtRS [27] and confirms the involvement of MtRS in telomere length maintenance.

We also tested other modalities of mitochondrial dysfunction such as shRNA mediated knock down of cytochrome c oxidase subunit IVi1 (CcO IVi1) and ATPase A6 mutation which was previously shown to affect mitochondrial oxidative phosphorylation [23,42]. First, a cybrid cell line harboring the m8993T>G mutation in the ATP Synthase 6 gene, with reduced state III respiration and reduced ADP/O ratio [43]. Second, Cytochrome c Oxidase subunit IVi1 (CcOIVi1) silenced C2C12 cells which showed activation of calcineurin mediated MtRS and phenotypic changes in different cells [23]. The ATP6 cybrid cell line exhibited 2-fold higher mRNA levels for hnRNPA2 and 20% shorter telomeres than the 143BTK- parental cells (Fig 3B). Similarly, CcOIVi1 silenced C2C12 cells and HEK293T cells showed significant telomere shortening and induction of MtRS genes CnAα and hnRNPA2 (Fig 3C). Our results suggest that mitochondrial dysfunction by depletion of mtDNA, pathogenic mtDNA mutations or disruption of CcO complex activates MtRS and induces telomere attrition. Oxidative stress, and attendant ROS has been shown to be one of the initiating signals in response to some modes of mitochondrial stress in some cell types [44]. Notably in agreement with our previous reports, induction of mitochondrial dysfunction either by mtDNA depletion or CcOIVi1 shRNA did not result in significant changes in mitochondrial ROS levels in the C2C12 cells or 293T cells used in this study. However, in cells that induced mitochondrial ROS by these treatment modalities also we observed activation of calcineurin and other MtRS markers (S3 Fig, and unpublished data).

### Telomere binding and acetylation of histones are mediated by the RNA binding domain of hnRNPA2

**Fig 4:**
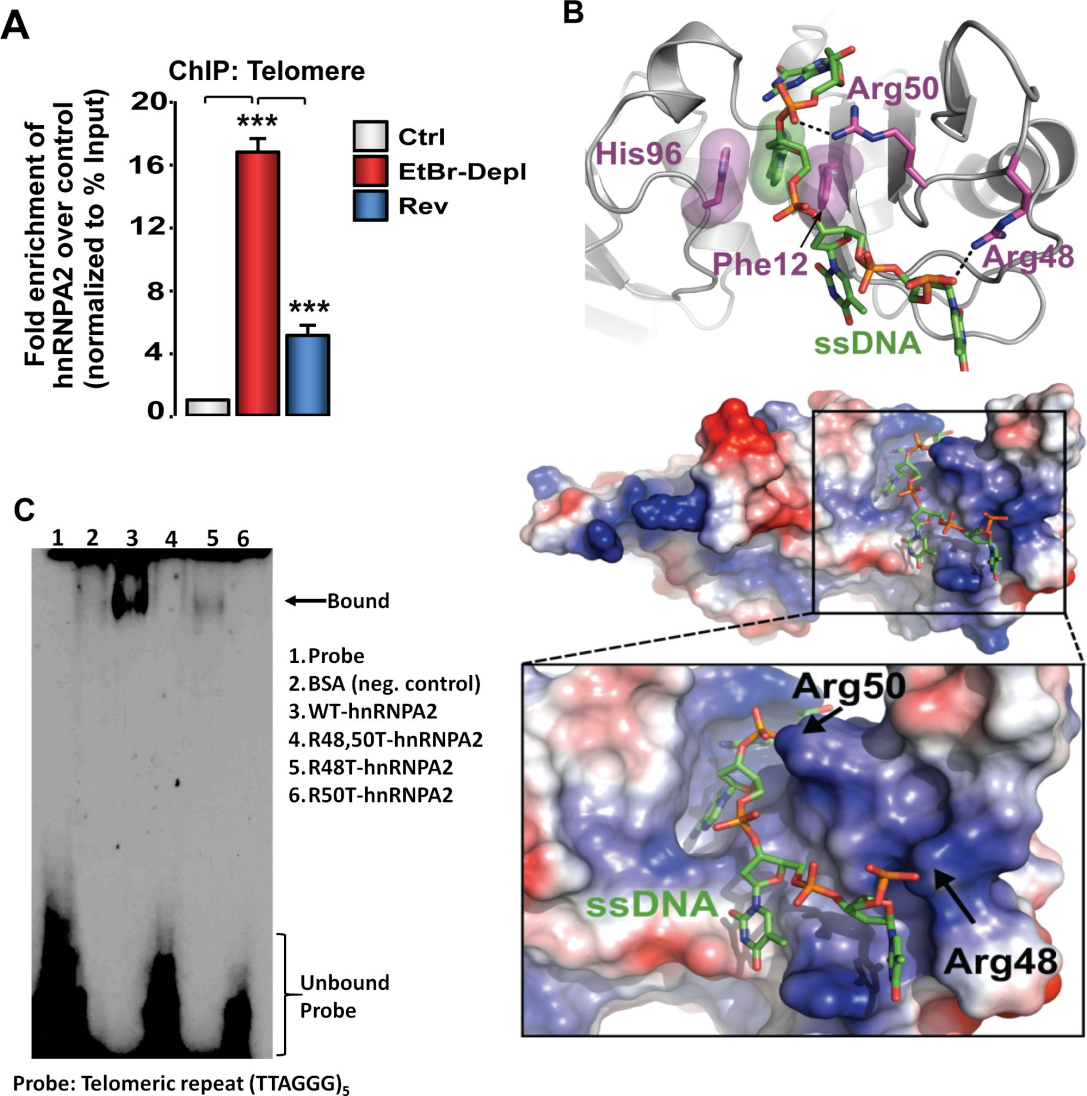
Identification of hnRNPA2 –telomere DNA binding domain. **(A)** Association of hnRNPA2 at telomeric DNA assessed by ChIP assay in control, MtDNA-depleted, and MtDNA-depleted/hnRNPA2sh C2C12 cells using hnRNPA2 antibody. **(B)** Model of hnRNPA2 in complex with single stranded telomere DNA. The hnRNPA2 model has a basic groove postulated to bind ssDNA (green), with a clear pocket for the adenosine ring and key interactions with Arg48 and Arg50. The electrostatic surface potential map was generated with Delphi [47] and is colored from -7 kTe-1 (blue) to +7 kTe-1 (red). **(C)** EMSA showing telomere binding of hnRNP proteins using purified hnRNPA2 WT as well as hnRNPA2 KAT mutant proteins.

HnRNPA2, a single strand RNA/DNA binding protein also binds to single-stranded telomeric repeat sequence [45,46]. In ChIP assays, we observed a 15-fold enrichment of hnRNPA2 at the telomeres of mtDNA-depleted cells, which was markedly reduced in reverted cells with substantially higher mtDNA content (~80% of control). These results suggest that mitochondrial dysfunction results in higher association of hnRNPA2 with the telomeric DNA (Fig 4A). Increased association of hnRNPA2 with telomeric DNA in mtDNA-depleted cells correlated with telomere shortening in these cells suggesting a possible correlation between hnRNPA2 binding and telomere shortening.

We generated a 3D model of the RNA binding domain (RBD) of hnRNPA2 bound to ssDNA based on the structure of hnRNPA1. In the ssDNA bound hnRNPA2 model (Fig 4B), one of the adenine bases in the ssDNA is stabilized by π-stacking interactions with His96 and Phe12, while Arg48 and Arg50 both form hydrogen bonds with phosphates in the DNA backbone. The RBDs of hnRNPA2 and hnRNPA1 are highly conserved, and thus the ssDNA binding mode in our model is similar to the one observed in the crystal structure of hnRNPA1 bound to ssDNA. The hnRNPA1: DNA complex (PDBID 1U1Q) shows that the protein establishes the majority of its specific interactions with an adenosine residue that is equivalent to the adenosine stabilized by His96 and Phe12 in our model.

To test the proposed model suggesting the involvement of Arg 48 and Arg50 residues in telomere binding, we generated hnRNPA2 mutant cell lines in which Arg residues were mutated either individually (R48T or R50T) or simultaneously (R48T/R50T). In our previous study we reported that the purity of the recombinant 6xHis-hnRNPA2 proteins were similar [28] (Figure A in S5 File). The electromobility shift assay (EMSA) using these recombinant proteins in Fig 4C shows that wild type hnRNPA2 protein binds to the single stranded telomeric DNA, while the KAT mutant hnRNPA2 proteins bind with markedly lower affinity (Fig 4C) confirming the role of the RBD residues in single stranded DNA binding. In a parallel EMSA, we used commercially available hnRNPA1, a telomere binding protein, as a positive control to test the binding efficiency of recombinant purified hnRNPA2 (Figure D in S4 File).

**Fig 5:**
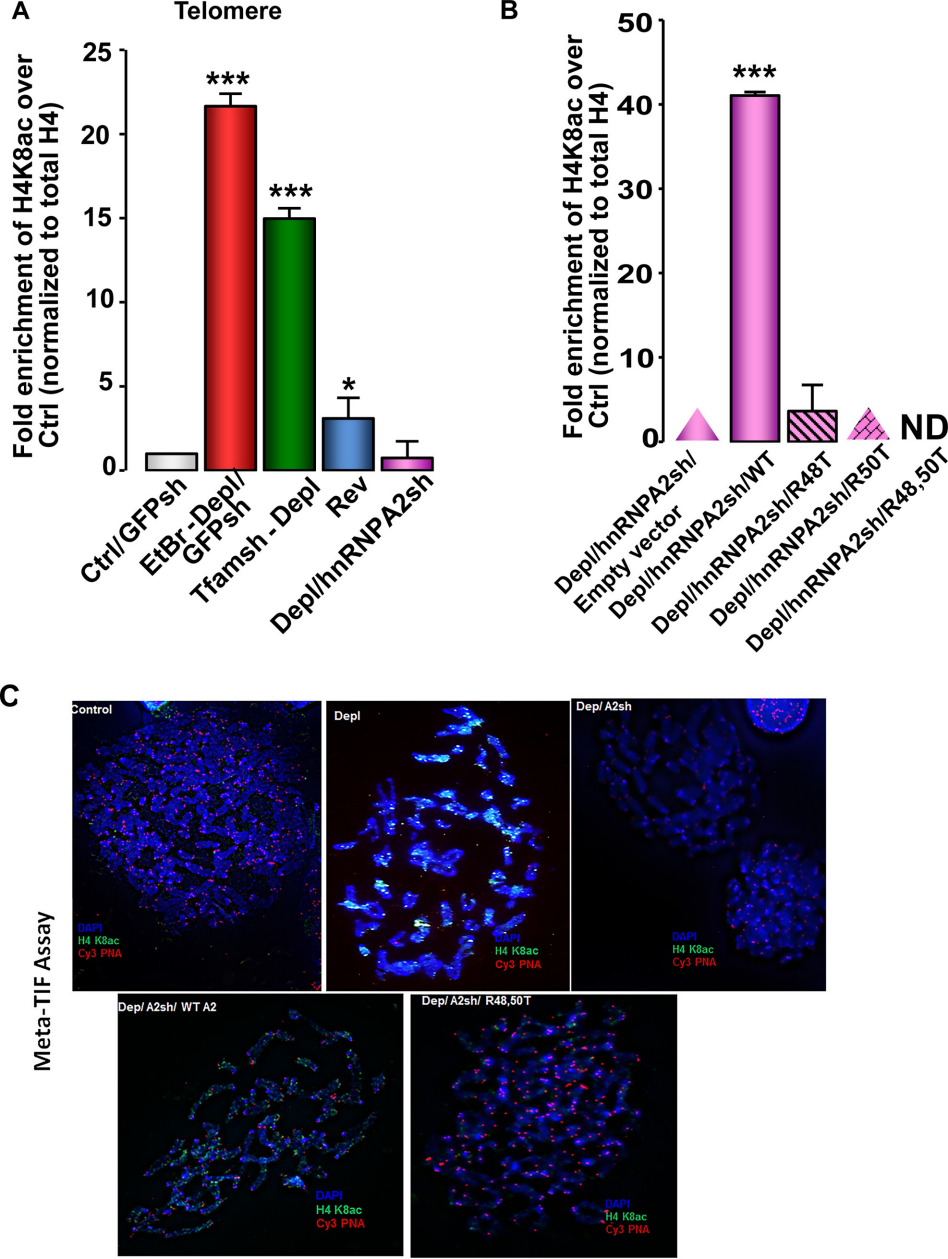
HnRNPA2-dependent telomere histone acetylation in MtDNA-depleted C2C12 cells. **(A)** ChIP assay in control, mtDNA-depleted, reverted and mtDNA-depleted/hnRNPA2sh cells showing enrichment of H4K8 acetylation using site-specific H4K8 acetylated antibody. **(B)** ChIP assays showing hnRNPA2 site specificity of telomeric histone H4K8 acetylation in mtDNA-depleted/hnRNPA2sh cells overexpressing hnRNPA2 WT and KAT mutants. **(C)** Meta-TIF assays in C2C12 cells stained with DAPI (metaphase chromosomes), Telomere-FISH (red), and H4K8Ac antibody IF (green).

The hnRNPA2 residues involved in acetyl-CoA binding [28] and telomeric ssDNA binding being identical, we investigated the contribution of two Arg residues R48 and R50 of hnRNPA2 in telomere dynamics. ChIP assay shows that telomeric DNA in mtDNA-depleted cells exhibited higher H4K8 acetylation, which was reduced in reverted cells and abrogated in mtDNA-depleted/hnRNPA2sh cells. These results demonstrate the involvement of hnRNPA2 in telomeric H4K8 acetylation (Fig 5A). We generated mtDNA depleted / hnRNPA2 shRNA cells expressing either the wild type or KAT mutant (R48T, R50T) cDNAs as described in materials and methods. We reported that while the mtDNA depleted / hnRNPA2 shRNA cells expressing wild type hnRNPA2 exhibited KAT activity, the R48T, R50T mutant expressing cells showed no KAT activity [28]. Cells reconstituted with the WT hnRNPA2 showed ~40-fold enrichment in the hnRNPA2 specific H4K8 acetylation, whereas H4K8 acetylation was undetectable in cells reconstituted with the hnRNPA2 KAT mutants (R48T, R50T) (Fig 5B). As we reported in our earlier study, the expression levels of hnRNPA2 were similar in the WT and KAT mutant expressing cells [28] (Figure B in S5 File). These results indicate that hnRNPA2 Arg48 and Arg50 residues are essential for H4K8 acetylation at the telomeres. These same residues were shown to be critical for hnRNPA2-AcCoA binding, hnRNPA2 mediated lysine acetyl transferase (KAT) activity [28]. It should be noted that in mtDNA depleted/hnRNPA2sh cells, we do not observe any telomere rescue which may seem contradictory. However, as stated above hnRNPA2 activates telomerase and mtDNA depleted/hnRNPA2sh cells lack the telomerase activation which is observed in mtDNA depleted cells (Figure C in S5 File). Therefore, we do not observe a rescue in telomere length in mtDNA depleted /hnRNPA2sh cells.

To confirm the relationship between telomere length and hnRNPA2 dependent telomeric acetylation we used an additional approach and performed Meta-TIF assay using a combination of *In Situ* hybridization of telomere specific Cy3-PNA probe (Telo-FISH) and staining with antibody (immunofluorescence) specific to the H4K8 acetylation (ab-IF) (Fig 5C). Our results show that in control C2C12 cells, there is undetectable telomeric H4K8 acetylation which has negative correlation with the relatively strong telomeric signal observed in these cells (Fig 5C). In mtDNA depleted cells, we observed abundant telomeric H4K8 acetylation (green) with a corresponding loss in telomeric signal (red). In agreement with our results above, the telomeric H4K8 acetylation is lost in the mtDNA depleted cells expressing hnRNPA2 shRNA suggesting that telomeric H4K8 acetylation is directly dependent on hnRNPA2 expression. Furthermore, mtDNA depleted and hnRNPA2 shRNA cells reconstituted with WT hnRNPA2 show telo-H4K8 acetylation while reconstitution with KAT mutant (R48T, R50T) hnRNPA2 did not show delectable telo-H4K8 acetylation further confirming the involvement of hnRNPA2 in telomeric histone acetylation. Results also show that R48 and R50 residues are important for this acetylation.

**Fig 6:**
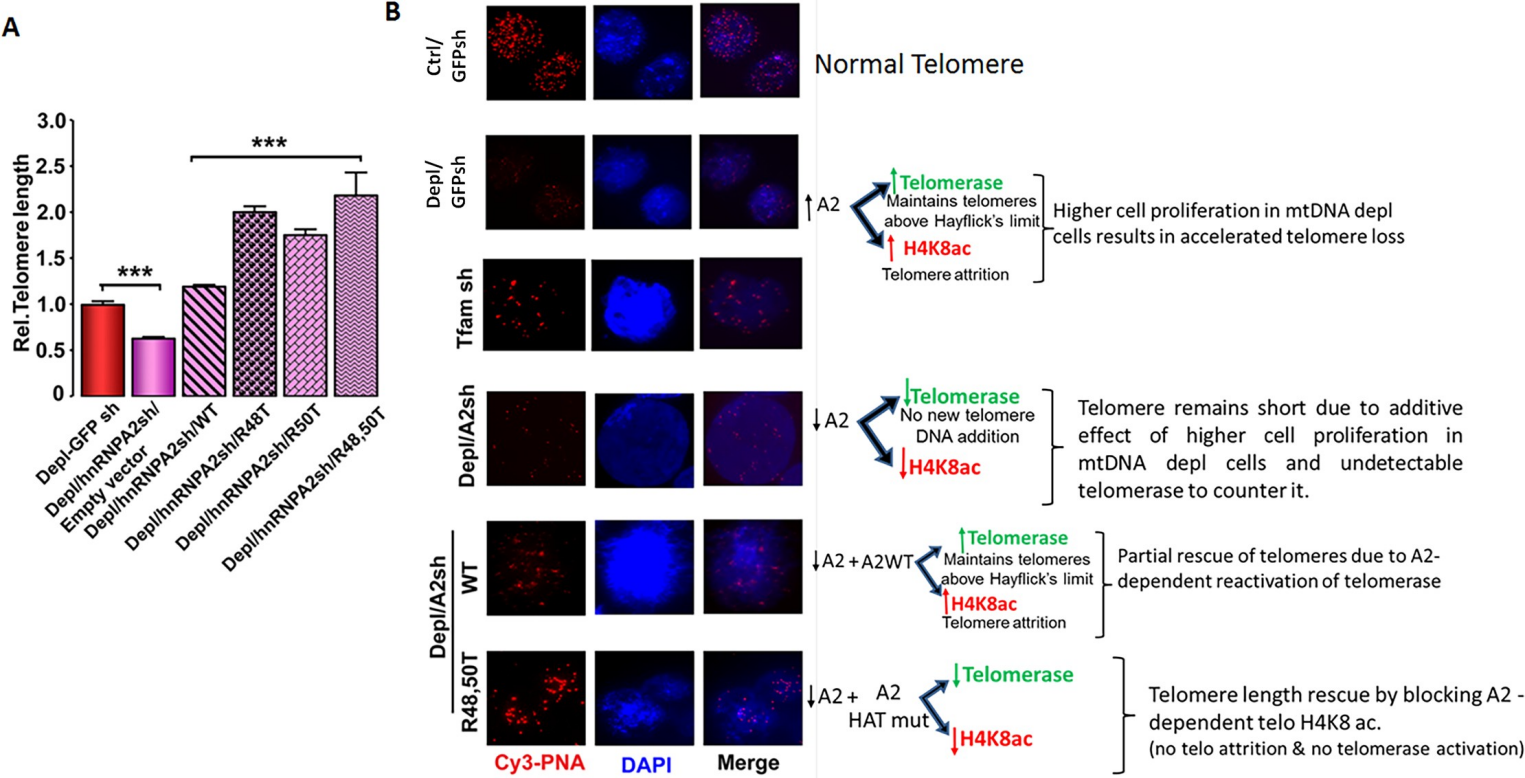
Regulation of telomere length by hnRNPA2-dependent telomere histone H4K8 acetylation in C2C12 cells. **(A)** Telomere length assessed by real time PCR in C2C12 mtDNA-depleted, mtDNA-depleted/hnRNPA2sh, mtDNA-depleted/hnRNPA2sh cells expressing WT and mutant hnRNPA2. **(B)** Q-FISH on nuclei of control, mtDNA-depleted, mtDNA-depleted / hnRNPA2shRNA and mtDNA-depleted / hnRNPA2sh cells expressing WT and mutant cells. Telomeres are probed with a Cy3-PNA telomere repeat probe (red) and nucleus stained with DAPI (blue). The width of each field shown is 15 μm.

Some studies suggest that telomere histone acetylation is a critical factor in telomere attrition [8,9]. Therefore, we investigated if the shortening of telomere length in response to mitochondrial dysfunction correlated with the hnRNPA2 mediated telomere H4K8 acetylation. Relative telomere length analysis using qPCR (Fig 6A and Figure C in S4 File) as well as telomere FISH analysis (Fig 6B) in different reconstituted cells showed that ectopic expression of WT hnRNPA2 in mtDNA-depleted/hnRNPA2sh C2C12 cells only marginally rescued the telomere length in mtDNA-depleted cells, while hnRNPA2 KAT mutants (R48T, R50T and the R48/50T double mutant) had more pronounced effects on rescuing telomere length (Fig 6 and S4 Fig). As we have reported previously [28] both Arg 48 and Arg 50 mutants, individually or together, diminish hnRNPA2 acetyltransferase activity and our results here show that these mutations also abrogate telomere histone H4K8 acetylation, and affect telomere length (Figs 4 and 5). We therefore infer that the loss of hnRNPA2 acetyltransferase activity likely underlies the rescue of telomere length (Fig 5). We ruled out the possibility that the rescue of telomere length observed in hnRNPA2 KAT mutant (R48T, R50T) cells is due to the increased binding of these mutant proteins to telomeric DNA because the EMSA data (Fig 4C) show that the mutant proteins bind to telomeric DNA probe very weakly.

## Discussion

Here we report an epigenetic mechanism by which mitochondrial dysfunction plays a role in inducing telomere attrition through acetyltransferase activity of hnRNPA2. Our results show that hnRNPA2 mediated H4K8 acetylation is a signal for telomere shortening. The importance of arginine 48 and 50 residues for hnRNPA2 acetyltransferase activity [28] is further confirmed by the markedly reduced telomeric H4K8 acetylation. Additionally we provide evidence that alterations in the telomere length in response to mitochondrial dysfunction is dependent on histone acetylation status because KAT mutant hnRNPA2 proteins, which show vastly reduced histone acetylation activity cause a rescue of telomere length. This is in agreement with previous reports in cancer cells where telomere histone acetylation has been shown to correlate with telomere length [8].

While our results show the causal role of mitochondrial dysfunction in telomere length maintenance by a novel epigenetic mechanism, prior studies have reported mitochondrial dysfunction as the result of telomere attrition [48–51], providing evidence for the close association between mitochondrial functions and telomere dynamics.

The consequence of telomere shortening in aging and cancer remains unclear. The prevailing view is that in aging, telomere shortening continues until cells senesce and finally die, while in cancer, the attrition stops when the telomere DNA reaches a critical length, and cells continue to divide and proliferate. It is suggested that induced telomerase activity in cancer cells is a critical factor, which prevents cell senescence and promotes tumor proliferation [52,53]. It may be seemingly contradictory that mitochondrial dysfunction in C2C12 and other immortalized cells induce a proliferative phenotype [25,54] in spite of mitochondrial stress induced telomere attrition. In a recent study tested the telomerase activity using a quantitative TRAP assay in these cell lines [28] (Figure C in S5 File) and found that mitochondrial stress induced signaling simultaneously activates telomerase in these cells. This provides a plausible explanation for these cells to maintain the critical telomere length for resisting senescence. In support, in IMR-90 cells, which do not express telomerase, we show that induction of mtDNA depletion results in hnRNPA2 activation and telomere shortening resulting in senescence. (Figure C in S3 File). Notably, in mtDNA-depleted/hnRNPA2sh C2C12 cells expressing hnRNPA2-KAT mutant proteins, the expression of the telomerase component TERT as well as telomerase activity was markedly low [28]. This suggests that the rescue of telomere length is possibly attributable to the reduced telomere histone acetylation but not due to activation of telomerase (Fig 6B). We also noted that hnRNP A2 silencing by shRNA expression did not rescue telomere length in mtDNA depleted cells. We attribute this to the absence of telomerase (expressed in mtDNA depleted cells) which also requires hnRNPA2 as transcription co-activator. In unpublished studies, we did not observed any significant difference in the formation of t-circles between control and mtDNA depleted C2C12 cells. The t-circles are elevated in homologous recombination-dependent (“ALT”) telomere maintenance. Therefore, in this study we have not considered the ALT as a possible mechanism for the observed telomere length reduction in our model.

It has been suggested that mtDNA depletion in cultured cells and animal models, causes altered nucleotide pools [55], which might be a plausible factor in telomere attrition and senescence. It is however noteworthy, that immortalized tumorigenic as well as non-tumorigenic cells, which also undergo altered nucleotide metabolism, continue to maintain telomeres at the critical length needed for survival and proliferation. We therefore believe that altered metabolic states induced by mitochondrial defects may not be the sole determining factor in telomere attrition. We cannot exclude the possibility that some of the attrition effect of telomere sequences is due to non-specific degradation.

Although, epigenetic regulation of gene expression by alterations in metabolism has been previously proposed [56,57] and mitochondrial translocation of TERT protein TIN2 has been reported to inhibit complex V activity (ATP5A1) and impair ATP synthesis [58], this is the first report demonstrating that mitochondrial stress signaling (MtRS) regulates epigenetic events, by histone acetylation on the telomeric chromatin. While our results here indicate that histone acetylation attributes to the loss of telomeric DNA, other possible mechanisms remain to be explored. Furthermore, we observed “fragile” ends in mtDNA-depleted cells, suggesting that MtRS potentially causes breakage of replication fork within telomeres [34]. We postulate that such cells either become senescent or apoptotic or that they bypass damage check points and go into proliferative mode. Our findings highlight the significance of mitochondrial dysfunction-induced MtRS as a common epigenetic link for telomere maintenance through the action of a common acetyltransferase protein, hnRNPA2 in aging and cancer cells.

## Supporting information

S1 Fig MtDNA depletion by silencing *Tfam* mRNA in IMR-90 cells. **(A)** Real time PCR showing relative mRNA levels of *Tfam* in IMR-90 cells expressing shRNA against either GFP (negative control) or *Tfam* (left panel). Relative mtDNA content assessed from total DNA by real time PCR using primers for mtDNA coded gene (COXI) or nuclear coded single copy gene (CcOIVi1) in IMR-90 cells expressing *Tfam* shRNA compared to the negative control cells expressing *GFP* shRNA (right panel). **(B)** Real time PCR analysis showing relative mRNA levels (compared to control cells) of retrograde signaling marker genes in *Tfam* shRNA expressing IMR-90 cells. Beta actin was used as endogenous control for normalization. **(C)** Total DNA (digested with Hinf I / Rsa I) from different cells (as indicated) was run on 0.8% agarose gel followed by in-gel Southern Hybridization using P-32 labeled telomere DNA probe. ***Left panel*** shows the ethidium bromide stained gel used for normalizing the total DNA content and autoradiograph on the right shows the amount of telomere DNA as indicated in the figure. Two DNA ladders: NEB 1KB Ladder and Lambda DNA-EcoR1/HindIII digest are loaded for size analysis. Lanes labeled 1–3 are control DNA samples (1μg) for Hinf1/Rsa1 digestion. This method allows estimation of total mass of telomere DNA, which is also an estimate of telomere length, under each treatment condition. ***Right panel*** shows the densitometry analysis showing total telomere mass in C2C12 cells normalized to the total DNA in each sample.
(PDF)

S2 Fig Zeocin induced DNA damage in C2C12 cells. (A) C2C12 cells were treated with Zeocin (as indicated in the figure) and telomere length (**B)** hnRNPA2 mRNA (*Right Panel*) were estimated by real time PCR. Data are represented as mean ± SD. **(C)** mtDNA was depleted in IMR-90 cells by *Tfam* shRNA expression or 2,3′-ddC treatment (10μM, 72h). **Left:** Cellular senescence analyzed by SA-β-galactosidase staining in parental and mtDNA depleted (either Tfam shRNA-expressing or 2,3′-ddC treated) IMR-90 cells. Panels depict different cell densities. Right: Quantitation of the SA-β-gal positive cells.
(PDF)

S3 Fig ROS levels in response to mitochondrial dysfunction. ROS production measured by relative DCF fluorescence in control, mtDNA-depleted, reverted and CcOIVi1shRNA C2C12 cells (*Left Panel*); control and CcOIVi1shRNA expressing HEK293 and TE11 cells (*Right Panel*). Cells were incubated with 1μM DCFDA and MitoCP (1μM) was added 3h before DCFDA addition. SOD-Catalase was added as a negative control. Data are represented as mean ± SD.
(PDF)

S4 Fig Telomere Q-PNA-FISH on C2C12 cell lines. **(A)** Telomere Q-FISH on nuclei of MtDNA-depl/hnRNPA2sh cells expressing hnRNPA2 KAT mutants. Telomeres are probed with Cy3-PNA (red) and nuclei stained with DAPI (blue). **(B)** Quantitation of the telomere signal intensities of at least 10 representative nuclei of each cell type. Telomere Cy3 signal intensity (Red) was normalized to the DAPI signal intensity (Blue) for each nucleus. Data are represented as mean ± SD. **(C)** Relative Telomere Length in control, mtDNA depleted cells, mtDNA-depleted/hnRNPA2sh and mtDNA-depleted/hnRNPA2sh cells expressing the WT and hnRNPA2 KAT mutants. **(D)** EMSA showing the binding efficiency of purified recombinant hnRNPA2 and hnRNPA1 to telomere DNA.
(PDF)

S5 Fig (Adapted from Guha et al Cell Discovery 2016 Dec 6;2:16045). **(A)** Coomassie Blue stained SDS-PAGE gel profile of bacterially expressed and purified recombinant 6xhis-hnRNPA2 wild type and KAT (R48T, R50T, R48T and R50T) mutants. **(B)** Western immunoblot showing hnRNPA2 levels in mtDNA depleted/ hnRNPA2shRNA cells expressing wild type and KAT mutant cDNAs **(C)** Real-time quantitative telomeric repeat amplification protocol (Q-TRAP) assay for the detection of telomerase activity in C2C12 cells.
(PDF)
