# Supplementary figures and images for: hnRNPA2 mediated acetylation reduces telomere length in response to mitochondrial dysfunction
Source: PLoS One. 2018 Nov 14;13(11):e0206897. doi: 10.1371/journal.pone.0206897 (PMC6241121; doi:10.1371/journal.pone.0206897)

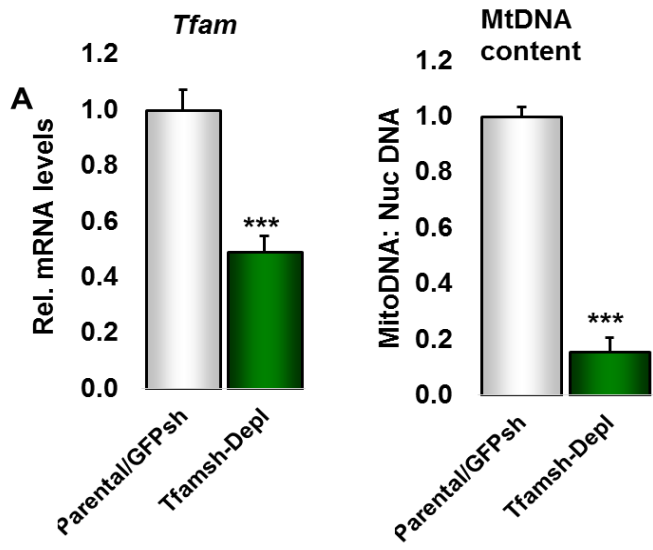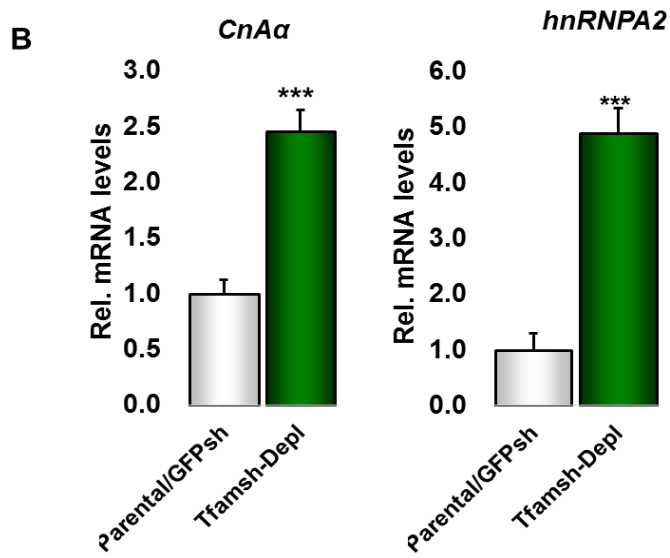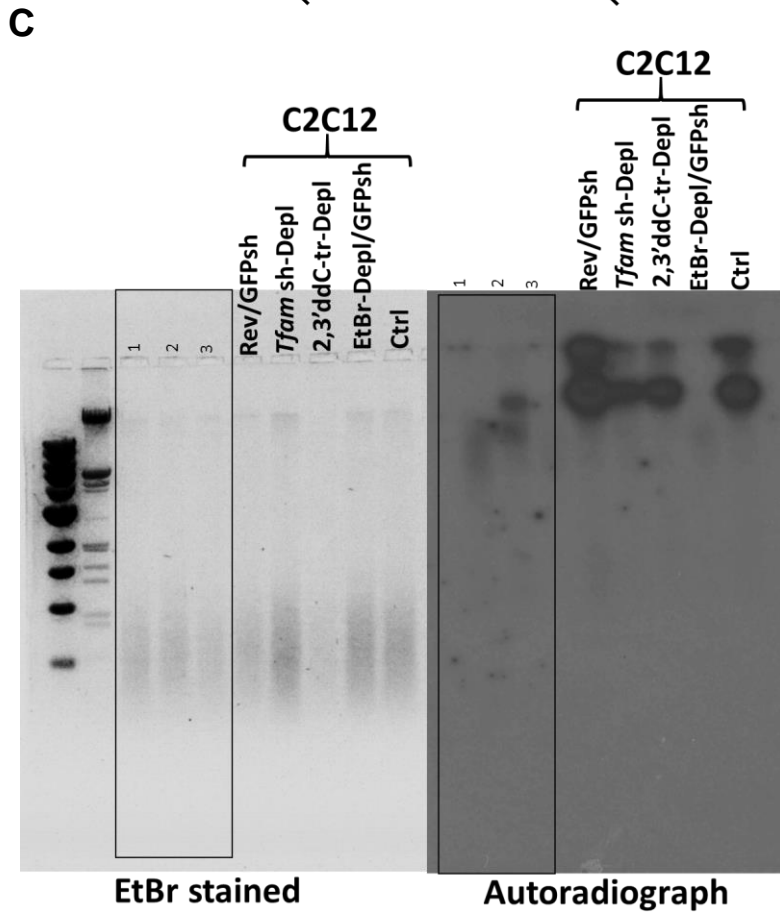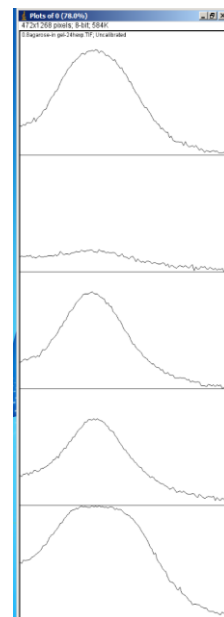

Supplement: S1 Fig — (A) Real time PCR showing relative mRNA levels of Tfam in IMR-90 cells expressing shRNA against either GFP (negative control) or Tfam (left panel). Relative mtDNA content assessed from total DNA by real time PCR using primers for mtDNA coded gene (COXI) or nuclear coded single copy gene (CcOIVi1) in IMR-90 cells expressing Tfam shRNA compared to the negative control cells expressing GFP shRNA (right panel). (B) Real time PCR analysis showing relative mRNA levels (compared to control cells) of retrograde signaling marker genes in Tfam shRNA expressing IMR-90 cells. Beta actin was used as endogenous control for normalization. (C) Total DNA (digested with Hinf I / Rsa I) from different cells (as indicated) was run on 0.8% agarose gel followed by in-gel Southern Hybridization using P-32 labeled telomere DNA probe. Left panel shows the ethidium bromide stained gel used for normalizing the total DNA content and autoradiograph on the right shows the amount of telomere DNA as indicated in the figure. Two DNA ladders: NEB 1KB Ladder and Lambda DNA-EcoR1/HindIII digest are loaded for size analysis. Lanes labeled 1–3 are control DNA samples (1μg) for Hinf1/Rsa1 digestion. This method allows estimation of total mass of telomere DNA, which is also an estimate of telomere length, under each treatment condition. Right panel shows the densitometry analysis showing total telomere mass in C2C12 cells normalized to the total DNA in each sample. (PDF) [file pone.0206897.s001.pdf]

C2C12 cells

A

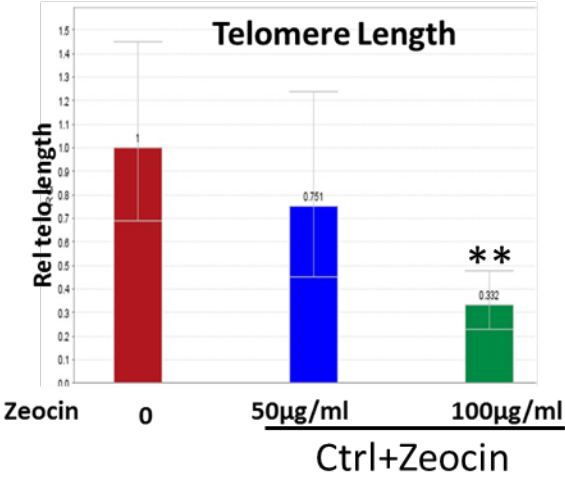

B

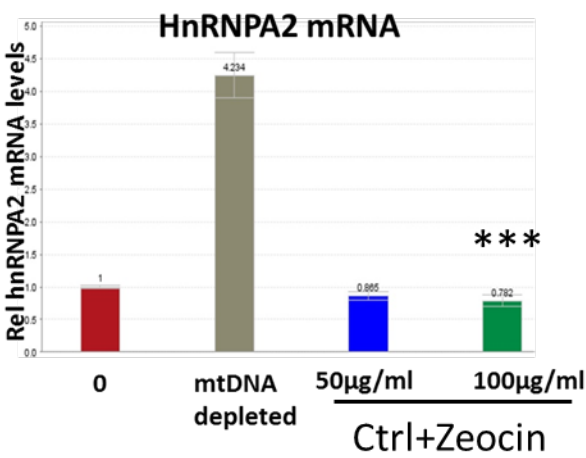

C

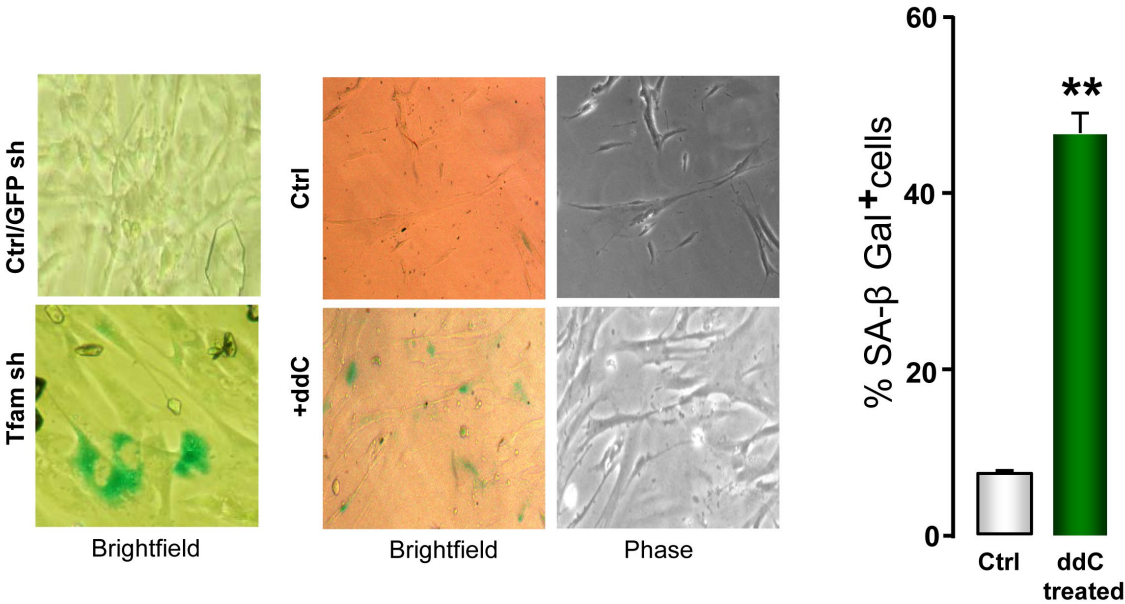

Supplement: S2 Fig — (A) C2C12 cells were treated with Zeocin (as indicated in the figure) and telomere length (B) hnRNPA2 mRNA (Right Panel) were estimated by real time PCR. Data are represented as mean ± SD. (C) mtDNA was depleted in IMR-90 cells by Tfam shRNA expression or 2,3′-ddC treatment (10μM, 72h). Left: Cellular senescence analyzed by SA-β-galactosidase staining in parental and mtDNA depleted (either Tfam shRNA-expressing or 2,3′-ddC treated) IMR-90 cells. Panels depict different cell densities. Right: Quantitation of the SA-β-gal positive cells. (PDF) [file pone.0206897.s002.pdf]

A

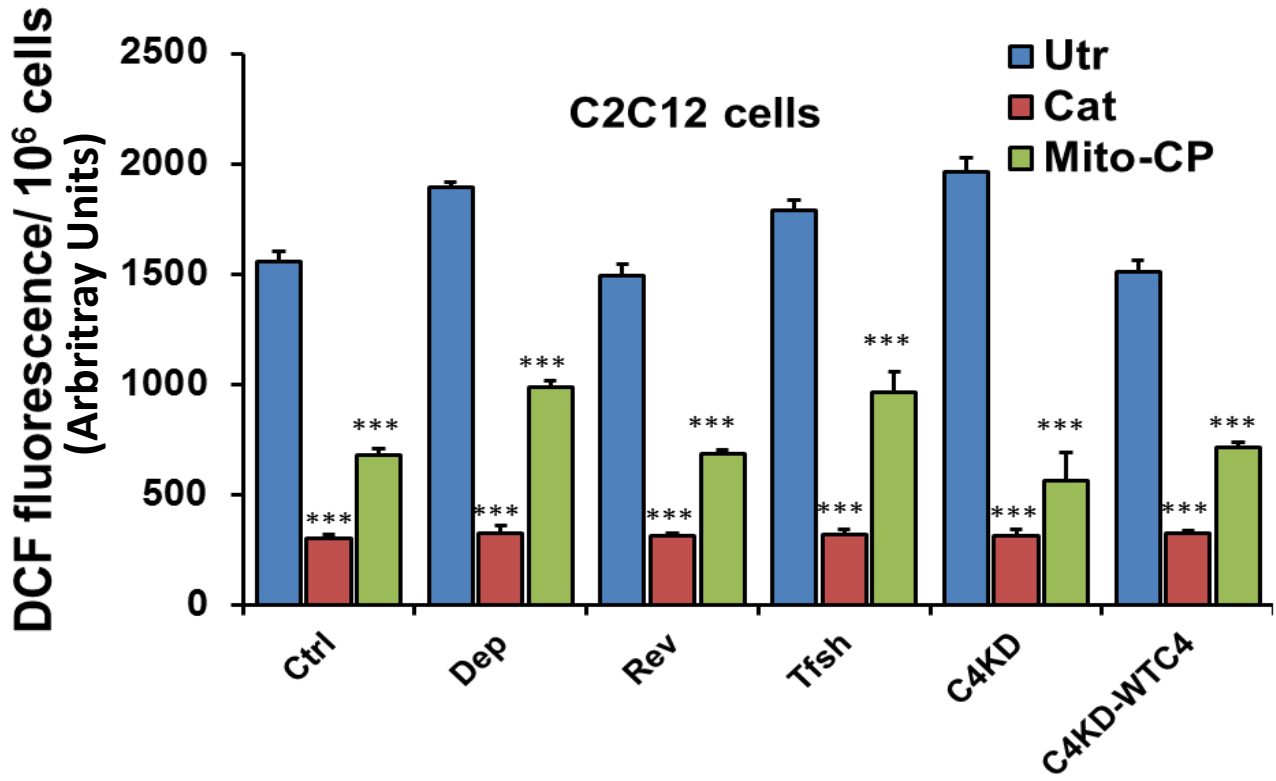

B

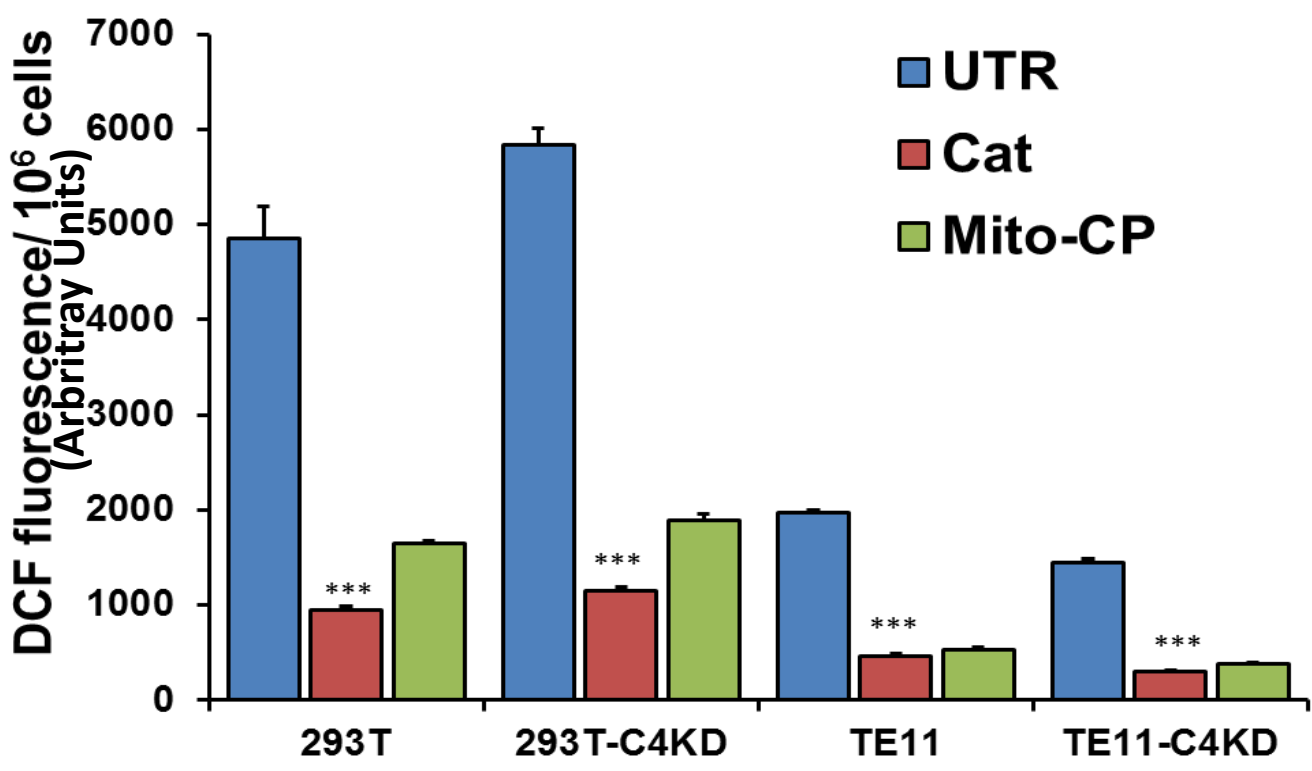

Supplement: S3 Fig — ROS production measured by relative DCF fluorescence in control, mtDNA-depleted, reverted and CcOIVi1shRNA C2C12 cells (Left Panel); control and CcOIVi1shRNA expressing HEK293 and TE11 cells (Right Panel). Cells were incubated with 1μM DCFDA and MitoCP (1μM) was added 3h before DCFDA addition. SOD-Catalase was added as a negative control. Data are represented as mean ± SD. (PDF) [file pone.0206897.s003.pdf]

A

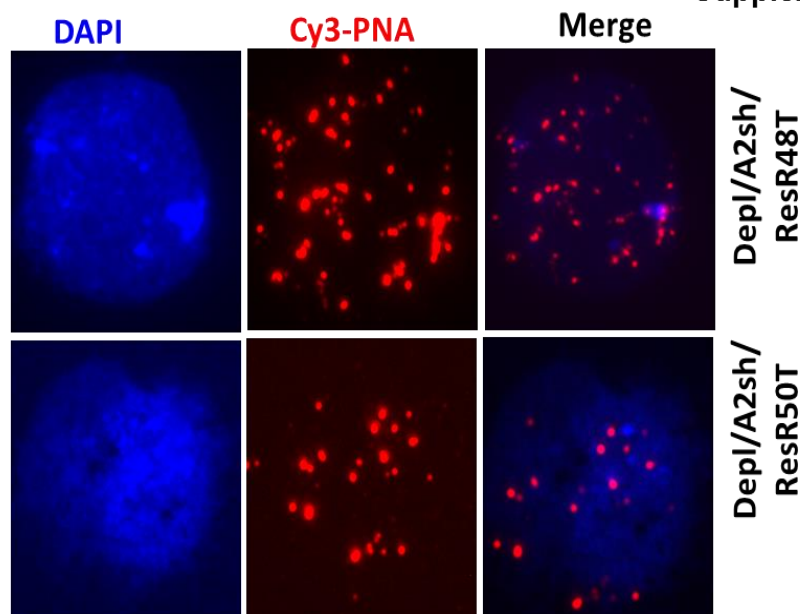

B

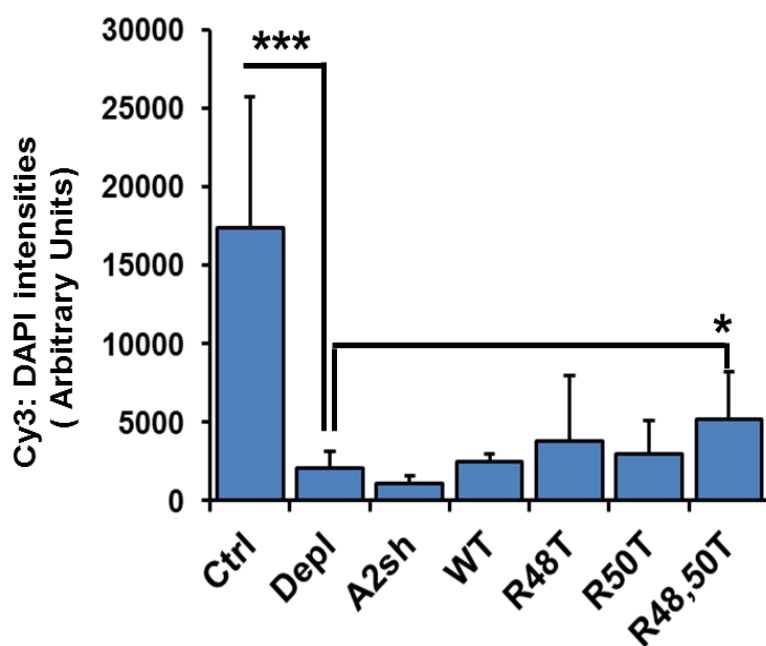

C

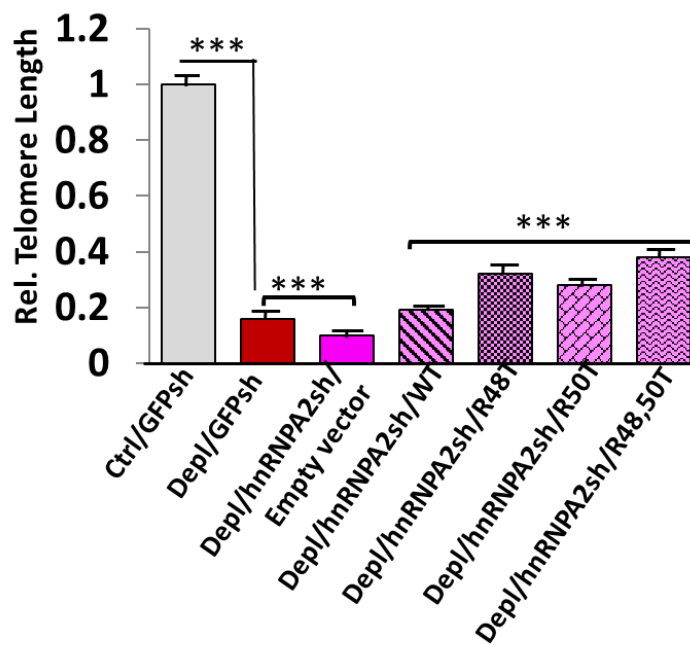

D

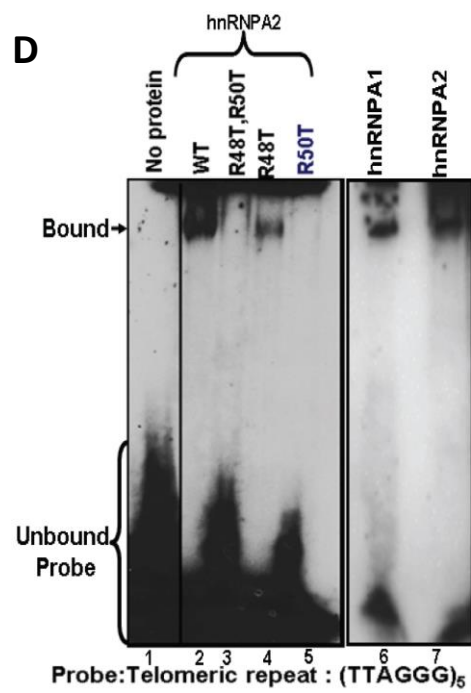

Supplement: S4 Fig — (A) Telomere Q-FISH on nuclei of MtDNA-depl/hnRNPA2sh cells expressing hnRNPA2 KAT mutants. Telomeres are probed with Cy3-PNA (red) and nuclei stained with DAPI (blue). (B) Quantitation of the telomere signal intensities of at least 10 representative nuclei of each cell type. Telomere Cy3 signal intensity (Red) was normalized to the DAPI signal intensity (Blue) for each nucleus. Data are represented as mean ± SD. (C) Relative Telomere Length in control, mtDNA depleted cells, mtDNA-depleted/hnRNPA2sh and mtDNA-depleted/hnRNPA2sh cells expressing the WT and hnRNPA2 KAT mutants. (D) EMSA showing the binding efficiency of purified recombinant hnRNPA2 and hnRNPA1 to telomere DNA. (PDF) [file pone.0206897.s004.pdf]

Supplemental Figure 5

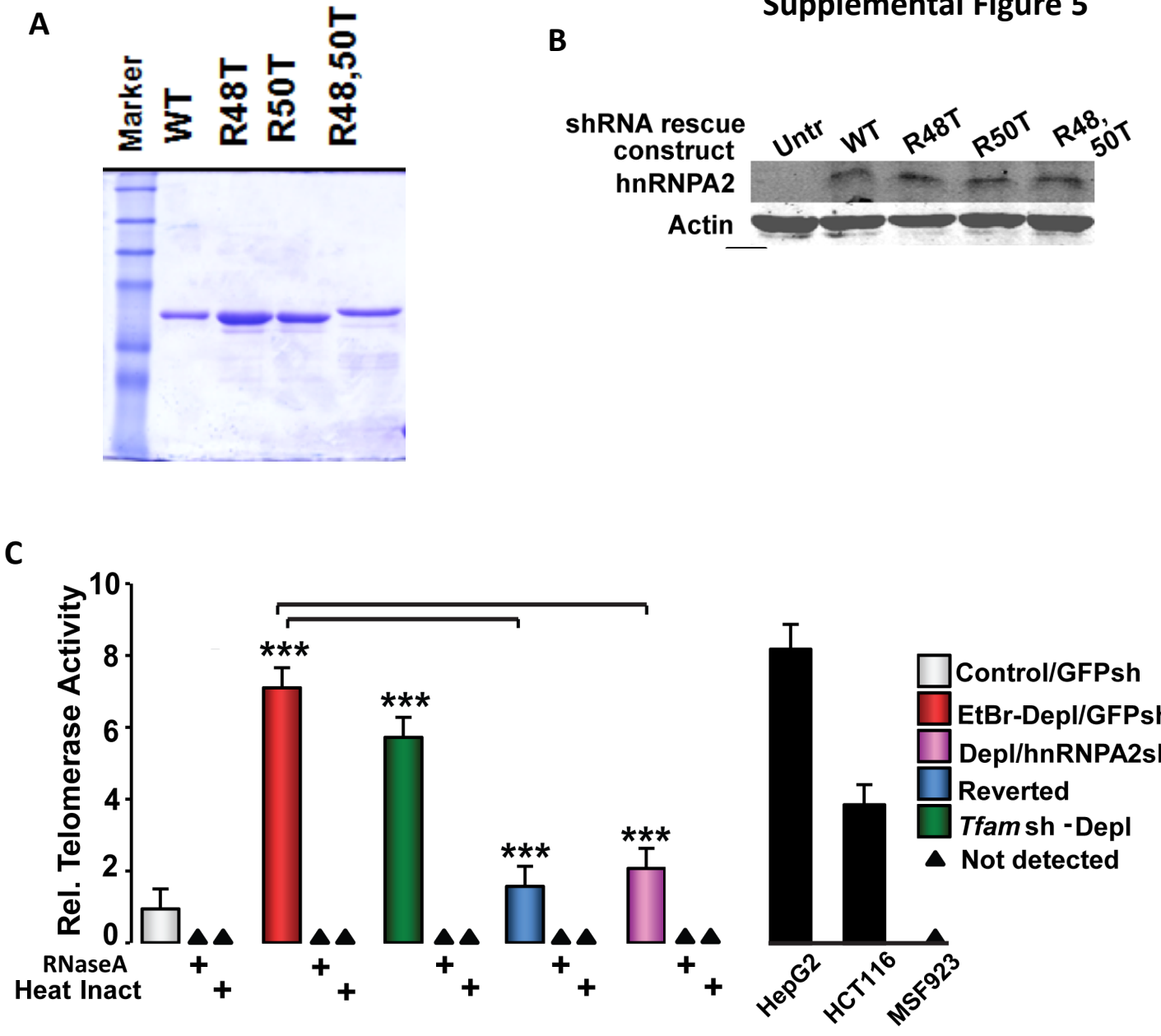

Adapted from Guha et al Cell Discov. 2016 Dec 6;2:16045.

Supplement: S5 Fig — (A) Coomassie Blue stained SDS-PAGE gel profile of bacterially expressed and purified recombinant 6xhis-hnRNPA2 wild type and KAT (R48T, R50T, R48T and R50T) mutants. (B) Western immunoblot showing hnRNPA2 levels in mtDNA depleted/ hnRNPA2shRNA cells expressing wild type and KAT mutant cDNAs (C) Real-time quantitative telomeric repeat amplification protocol (Q-TRAP) assay for the detection of telomerase activity in C2C12 cells. (PDF) [file pone.0206897.s005.pdf]
